# Ten-year outcomes of high-dose intensity-modulated radiation therapy for nonmetastatic prostate cancer with unfavorable risk: early initiation of salvage therapy may replace long-term adjuvant androgen deprivation

**DOI:** 10.1007/s10147-019-01478-y

**Published:** 2019-05-31

**Authors:** Rihito Aizawa, Kenji Takayama, Kiyonao Nakamura, Takahiro Inoue, Toshinari Yamasaki, Takashi Kobayashi, Shusuke Akamatsu, Osamu Ogawa, Takashi Mizowaki

**Affiliations:** 1 Department of Radiation Oncology and Image-Applied Therapy, Graduate School of Medicine, Kyoto University, 54 Shogoin Kawahara-cho, Sakyo-ku, Kyoto, 606-8507 Japan; 2 Department of Urology, Graduate School of Medicine, Kyoto University, 54 Shogoin Kawahara-cho, Sakyo-ku, Kyoto, 606-8507 Japan

**Keywords:** Prostate cancer, Unfavorable risk, Intensity-modulated radiation therapy, Short-term androgen deprivation therapy, Early salvage androgen deprivation therapy

## Abstract

**Background:**

The optimal timing of salvage androgen deprivation therapy (ADT) following definitive radiation therapy for prostate cancer (PCa) is unknown. This study evaluated the efficacy of early initiation of salvage-ADT (S-ADT) based on predetermined timing among patients with unfavorable PCa treated with high-dose intensity-modulated radiation therapy (IMRT).

**Materials and methods:**

High-risk (HR) and very-high-risk (VHR) PCa patients treated with IMRT at our institution between September 2000 and December 2010 were analyzed retrospectively. Treatment consisted of high-dose IMRT (78 Gy/39 fractions) combined with 6 months of neoadjuvant-ADT (NA-ADT). S-ADT was initiated when prostate-specific antigen levels exceeded 4.0 ng/mL.

**Results:**

In total, 268 (184 HR and 84 VHR) patients were analyzed. The median follow-up period was 114.4 months. The 10-year overall survival (OS), PCa-specific survival (PCSS), biochemical failure (BF), and clinical failure (CF) rates were 82.8%, 97.1%, 27.3%, and 12.8% among the HR PCa patients and 79.4%, 87.9%, 56.2%, and 26.7% among the VHR PCa patients (*p* = 0.839, = 0.0377, < 0.001, and < 0.001), respectively. The 10-year cumulative incidence rates of urinary and rectal (grades 2–3) toxicities were 22.6% and 5.8%, respectively. No grade 4 or higher toxicities were observed.

**Conclusion:**

High-dose IMRT combined with short-term NA-ADT resulted in long-term disease-free status, with acceptable morbidity among approximately three-fourths of the HR PCa patients and nearly half of the VHR PCa patients. Moreover, excellent survival outcomes were achieved by the early S-ADT initiation. This approach may be a promising alternative to uniform provision of long-term ADT.

## Introduction

External beam radiotherapy (EBRT) is a major treatment modality for nonmetastatic prostate cancer (PCa). Intensity-modulated radiation therapy (IMRT) allows the radiation dose to be increased safely by selectively protecting a significant volume of the rectum from high-dose radiation, facilitating its widespread clinical use.

The standard approach for unfavorable PCa is high-dose EBRT combined with long-term androgen deprivation therapy (ADT) for 2–3 years [1]. However, the optimal duration of ADT combined with high-dose EBRT remains unclear, because this evidence is based on results using 70 Gy or lower via three-dimensional conformal radiotherapy (3D-CRT) [2-6]. Up to now, no mature result of high-dose IMRT for unfavorable PCa is available from previous reports. In addition, the optimal timing of salvage-ADT (S-ADT) after disease failure has not been well-established, although the potential benefits of earlier S-ADT initiation have been suggested [7, 8]. To our knowledge, no prospective studies of unfavorable PCa have determined the timing of S-ADT initiation in advance. Furthermore, previous studies of S-ADT were mainly combined with long-term adjuvant-ADT (A-ADT), which may have masked the effect of S-ADT. Therefore, the true benefit of early S-ADT induction after disease failure following definitive EBRT remains unclear.

In the present study, we reported the clinical significance of early S-ADT induction based on the predetermined timing for unfavorable PCa treated with 6 months of neoadjuvant-ADT (NA-ADT) and high-dose IMRT. Unlike previous studies, because no A-ADT was administered following IMRT, we were able to observe the direct effect of S-ADT without masking by A-ADT. Specifically, it may serve as a benchmark comparison with the uniform application of long-term A-ADT. To the best of our knowledge, this is the first report investigating the efficacy of early S-ADT initiation based on predetermined timing in combination with high-dose IMRT, with a long follow-up period (9.5-year) and a large cohort of patients (*n* = 268).

## Materials and methods

This study adhered to the tenets of the Declaration of Helsinki, with approval from our institutional ethical review board (approval No: R1048). Written informed consent was obtained from all patients.

### Patients

We retrospectively reviewed our prospectively maintained institutional PCa registry and searched for eligible patients. The eligibility criteria were as follows: (1) clinical T1-4N0M0 (according to the classification of the International Union Against Cancer 1997) adenocarcinoma of the prostate with high-risk (HR) or very-high-risk (VHR) features (National Comprehensive Cancer Network risk classification version 2, 2018) [1], (2) treatment with IMRT to the prostate and seminal vesicles (SVs) alone between September 2000 and December 2010 at our institution, (3) NA-ADT duration < 12 months, (4) prescription dose ≥ 74 Gy, and (5) no addition of adjuvant therapy. Patients with castration-resistant PCa (CRPC) at the initiation of IMRT were excluded. Initial evaluations included needle biopsies (usually ≥ 8 cores), digital rectal examinations, transrectal ultrasonography, computed tomography (CT), magnetic-resonance imaging, and bone scintigraphy (BS). All pathological specimens were re-evaluated at our institution.

### Neoadjuvant androgen deprivation therapy

Prior to IMRT, androgen suppression consisted of 6 months of combined androgen blockade (CAB) with a luteinizing hormone-releasing hormone (LH-RH) analogue and anti-androgenic agent. However, there were variations in the durations of the treatments because a number of patients were introduced to our hospital after ADT had been initiated. In addition, patients with liver dysfunction or special requests were administered the LH-RH analogue only.

### Intensity-modulated radiation therapy

The details of our IMRT protocol have been reported previously [9]. Briefly, a five-field dynamic multi-leaf collimator technique and 6–15-MV photon beams were used. The prostate and SVs (proximal two-thirds for non-T3b, whole for T3b) were treated. Elective pelvic irradiation was not performed. Set-up error correction was based on the pelvic bone structure. A total of 78 Gy in 39 fractions was prescribed, which was reduced by 4 Gy in patients with a risk factor for rectal bleeding, such as those with advanced age (≥ 80 years), anticoagulant/antiplatelet therapy, and severe diabetes mellitus. For patients with multiple risk factors for rectal bleeding, total dose was reduced by 8–12 Gy (those patients were excluded from this analysis).

### Patient follow-up and salvage therapy

No A-ADT was administered to any patient following IMRT. Prostate-specific antigen (PSA) levels were assessed every 1–3 months during the first 2 years, every 3–6 months thereafter. In selected patients with a stable clinical course, this interval was extended annually after 5 years. No additional radiographic study after IMRT was required, unless an increase in the PSA level or symptoms suggesting clinical failure (CF) was observed. Salvage therapy which comprised continuous or intermittent ADT was initiated when PSA levels exceeded 4.0 ng/mL in a monotonically increasing manner to eliminate false failure cases (biochemical failure [BF] without continuous PSA elevation). Before initiating salvage therapy, CT and BS were conducted.

Data collection (clinical outcomes and toxicities) was conducted using a follow-up data sheet at every visit and the prospectively maintained institutional database.

### Outcome evaluation and statistical analyses

The time of occurrence of each event was calculated from the date of IMRT initiation. The Kaplan–Meier method was used to estimate the overall survival (OS) and PCa-specific survival (PCSS) rates, and the log-rank test was used to compare the rates between the HR and VHR groups. To account for death without each event being a competing risk, the cumulative incidence method was used to estimate BF and CF rates, and Gray’s test was used to determine the differences between the HR and VHR groups. The BF was evaluated based on the Phoenix definition [10]. To identify potential factors affecting BF and CF, we performed univariate analysis (UVA) and multivariable analysis (MVA) using the Fine and Gray’s regression model. Factors included pretreatment PSA (iPSA) (> 30 vs. 20–30 vs. ≤ 20 ng/mL), clinical T stage (T3b–4 vs. T3a vs. T1–2), Gleason Score (GS) sum (≥ 8 vs. ≤ 7), cores with a GS sum of 8–10 (≥ 5 vs ≤ 4) and irradiation dose (74 Gy vs 78 Gy).

Acute urinary and rectal toxicities (within the first 90 days after IMRT initiation) and late urinary and rectal toxicities were evaluated based on the National Cancer Institute Common Toxicity Criteria, version 2. The cumulative incidence method was used to estimate the rates of late-radiation toxicities.

A value of *p* < 0.05 denoted statistical significance. All statistical analyses were performed using R version 3.1.1 (The R Foundation for Statistical Computing, Vienna, Austria).

## Results

### Patient characteristics

We identified 273 patients who met the eligibility criteria. Among them, pelvic lymph node surgical dissection was performed before IMRT in two patients, and bone or pelvic lymph node metastasis before IMRT was detected retrospectively in three patients. These patients were excluded, and the remaining 268 patients were included in the analyses.

The median patient age was 71 (interquartile range [IQR] 65–75) years at the initiation of IMRT. The median iPSA level was 20.8 (IQR: 13.2–36.5) ng/mL. More than half of the patients (*n* = 145) had a GS sum ≥ 8, and approximately 70% of the patients had ≥ T3a disease (*n* = 188); 184 and 84 patients were categorized into the HR and VHR groups, respectively. The patient characteristics are summarized in Table 1.

**Table 1:** Patient and treatment characteristics

Age (years)	
Median	71
IQR	65–75
Clinical T stage, *n* (%)	
T1c	24 (9.0)
T2a	24 (9.0)
T2b	14 (5.1)
T2c	18 (6.7)
T3a	135 (50.4)
T3b	48 (17.4)
T4	5 (1.9)
iPSA (ng/mL)	
Median	20.8
IQR	13.2–36.5
Gleason score, *n* (%)	
6	13 (4.9)
7	110 (41.0)
8	85 (31.7)
9	55 (20.5)
10	5 (1.9)
NCCN risk classification, *n* (%)	
High-risk	184 (68.7)
Very high-risk	84 (31.3)
NA-ADT, *n* (%)	
CAB	252 (94.0)
LH–RH	16 (6.0)
Duration of NA-ADT (months)	
Median	6.3
IQR	5.0–7.6
Salvage therapy, *n* (%)	87 (32.5)
PSA at initiation of salvage therapy (ng/mL)	
Median	5.5
IQR	4.2–6.6
IMRT	
Dose, *n* (%)	
78 Gy	208 (77.6)
74 Gy	60 (22.4)

*IQR* interquartile range, *iPSA* pretreatment prostate-specific antigen, *NCCNrisk classification* National Comprehensive Cancer Network risk classification ver. 2. 2018; *NA-ADT* neoadjuvant androgen deprivation therapy, *CAB* combined androgen blockade, *LH–RH* luteinizing hormone–releasing hormone analog, *IMRT* intensity-modulated radiation therapy

### Treatments

All patients received NA-ADT for a median duration of 6.3 (IQR 5.0–7.6) months. A total of 252 patients (94.0%) received CAB and the remaining 16 patients (6.0%) were treated with the LH–RH analogue alone. The median dose was 78 (IQR 74–78) Gy delivered in 39 (IQR 37–39) fractions. The full dose was delivered to 208 patients (77.6%) and the reduced dose was delivered to the remaining 60 patients (22.4%). The details of the treatments are summarized in Table 1.

### Oncological and survival outcomes

**Fig. 1:**
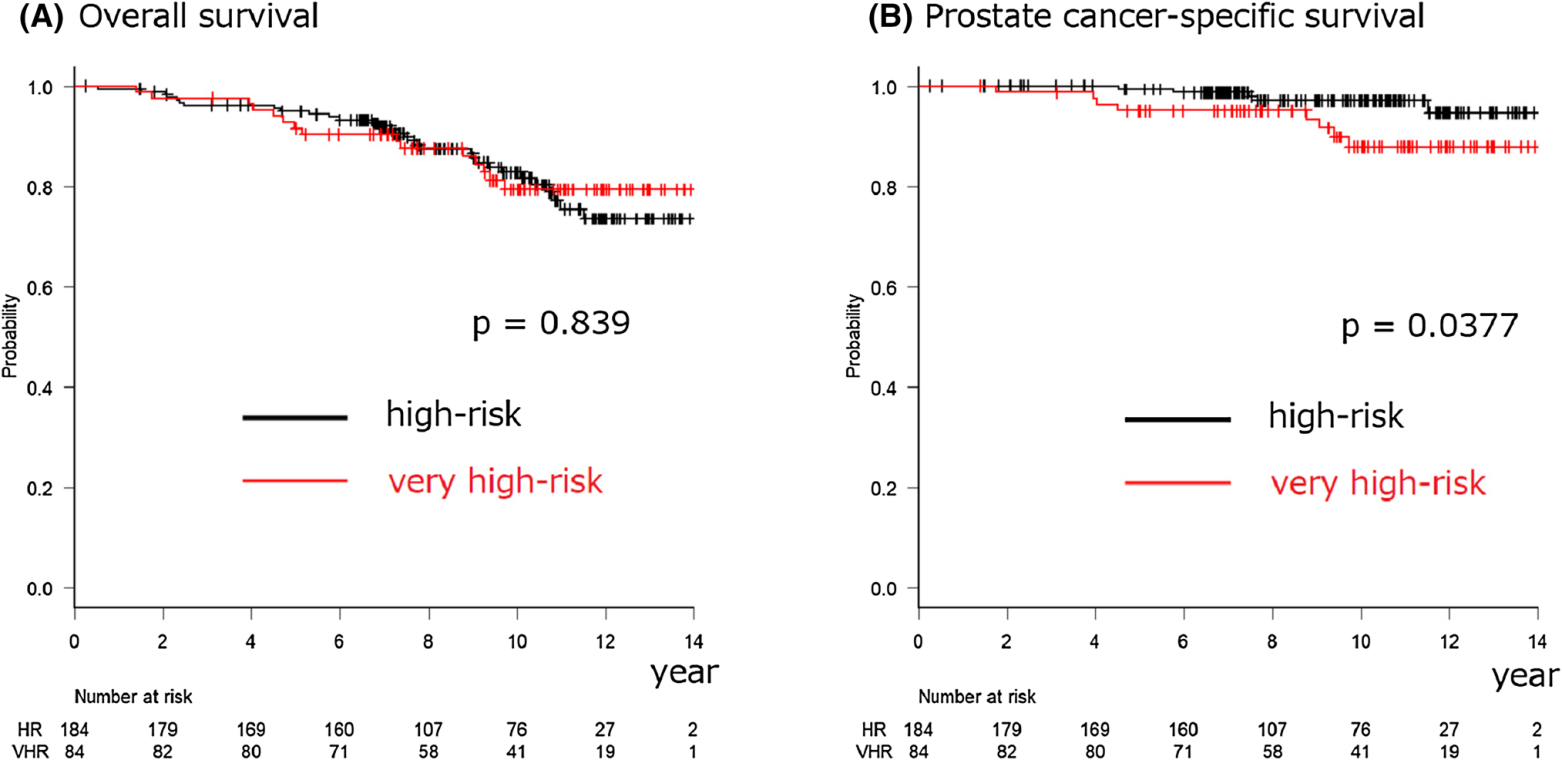
Kaplan–Meier curves for overall-survival (**a**) and prostate-cancer-specific-survival (**b**) rates after intensity-modulated radiation therapy according to the National Comprehensive Cancer Network risk classification

The median follow-up period was 114.4 (IQR 84.6–137.6) months. During follow-up, there were 46 deaths*.* In this group, nine patients died from PCa, while four patients were lost to follow-up with either progressive CRPC or the best supportive care. Therefore, all of these 13 patients were recorded as death from PCa. Among those 13 patients, the median times to BF, CF, and death after IMRT were 13.1 (IQR 11.0–25.2), 32.0 (IQR 17.3–63.4), and 89.5 (IQR 54.0–108.8) months, respectively. The characteristics of patients who died from PCa are summarized in Table 2. The 5- and 10-year OS rates were 95.0% (95% confidence interval [CI] 90.6–97.4) and 82.8% (95% CI 75.4–88.2) in the HR group and 91.6% (95% CI 83.2–95.9) and 79.4% (95% CI 68.0–87.2) in the VHR group, respectively (*p* = 0.839) (Fig. 1a). The 5- and 10-year PCSS rates were 99.4% (95% CI 95.9–99.9) and 97.1% (95% CI 92.4–98.9) in the HR group and 95.1% (95% CI 87.6–98.1) and 87.9% (95% CI 76.9–93.9) in the VHR group, respectively (*p* = 0.0377) (Fig. 1bss).

**Table 2:** The characteristics of patients who developed biochemical, clinical failure or death from prostate cancer

	Biochemical failure	Clinical failure	Prostate cancer death
Number of patients	97	45	13
Age (years)			
Median	69	68	67
IQR	63–73	61–71	62–75
Clinical T stage, *n* (%)			
T1c	8 (8.2)	2 (4.4)	1 (7.7)
T2a	5 (5.2)	2 (4.4)	0 (0)
T2b	3 (3.1)	1 (2.2)	0 (0)
T2c	5 (5.2)	4 (8.9)	0 (0)
T3a	45 (46.4)	20 (44.5)	6 (46.1)
T3b	27 (27.8)	13 (28.9)	4 (30.8)
T4	4 (4.1)	3 (6.7)	2 (15.4)
iPSA (ng/mL)			
Median	31.2	24.7	27.1
IQR	18.5–49.2	14.6–38.1	13.4–33.0
Gleason score, *n* (%)			
6	3 (3.1)	0 (0)	0 (0)
7	35 (36.1)	14 (31.1)	4 (30.8)
8	32 (33.0)	16 (35.5)	5 (38.4)
9	24 (24.7)	12 (26.7)	3 (23.1)
10	3 (3.1)	3 (6.7)	1 (7.7)
NCCN risk classification, *n* (%)			
High-risk	49 (50.5)	20 (44.4)	5 (38.5)
Very high-risk	48 (49.5)	25 (55.6)	8 (61.5)

**Fig. 2:**
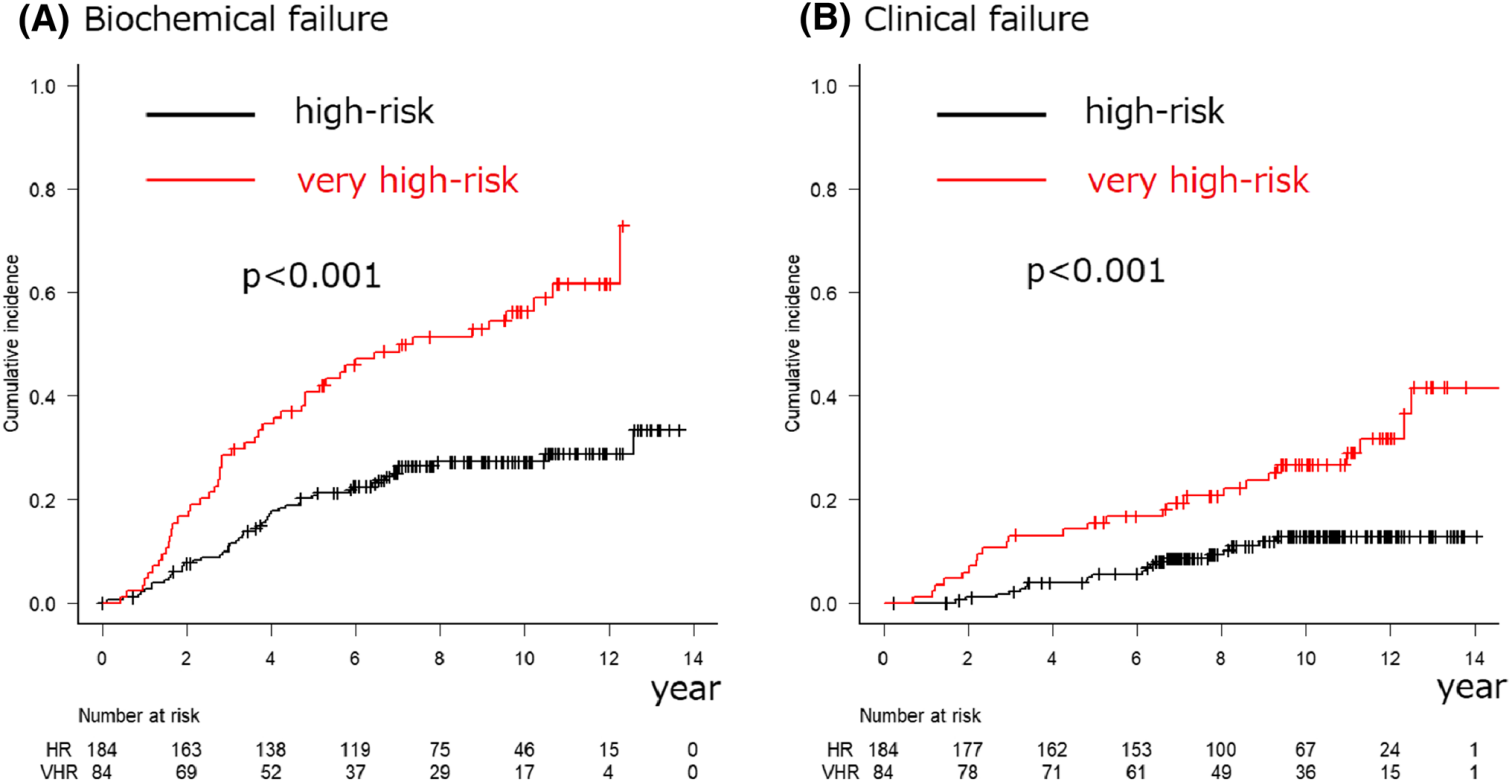
Cumulative incidence rates of biochemical failure (**a**) and clinical failure (**b**) after intensity-modulated radiation therapy according to the National Comprehensive Cancer Network risk classification

During follow-up, 97 patients developed BF, with a median period of 39.8 (IQR 22.3–62.2) months after IMRT. Among the patients who developed disease failure, the initial patterns of disease failure were BF in all cases. The characteristics of patients who developed BF are summarized in Table 2. The 5- and 10-year BF rates were 20.7% (95% CI 15.1–26.9) and 27.3% (95% CI 20.8–34.2) in the HR group and 40.7% (95% CI 30.1–51.1) and 56.2% (95% CI 44.2–66.6) in the VHR group, respectively (*p* < 0.001) (Fig. 2a).

During follow-up, 45 patients developed CF, with a median period of 59.3 (IQR 32.0–96.8) months after IMRT. In this group, CF was detected at or before the initiation of salvage therapy in 29 of the patients (castration-sensitive PCa), while CF was detected in 16 of the patients during salvage therapy (CRPC). The characteristics of patients who developed CF are summarized in Table 2. The 5- and 10-year CF rates were 5.6% (95% CI 2.9–9.7) and 12.8% (95% CI 8.0–18.8) in the HR group and 15.5% (95% CI 8.7–24.1) and 26.7% (95% CI 17.3–36.9) in the VHR group, respectively (*p* < 0.001) (Fig. 2b). Local failure was observed in eight patients during follow-up. Among them, additional local treatment was necessary for four patients during follow-up: transurethral resection of prostate and/or nephrostomy (*n* = 3) and salvage brachytherapy (BT) (*n* = 1).

Among the 97 patients who developed BF, salvage therapy was initiated in 87 patients (89.7%) due to continuous PSA elevation, with a median PSA of 5.48 (IQR 4.18–6.61) ng/mL at initiation of salvage therapy. Continuous ADT was initially used as salvage therapy in 31 of the patients. In this group, continuous ADT was discontinued or changed to an intermittent method in six of the patients with a median period of 44.7 (IQR 29.0–45.2) months after initiation of salvage ADT, owing to a favorable clinical course during the initial continuous ADT. No patient was treated with androgen receptor-axis-targeted agents or docetaxel in the castration-sensitive setting.

### Toxicities

Table 3 summarizes the acute and late toxicities. Acute urinary toxicities consisted mostly of urinary frequency, urgency, or retention. Grade 3 dysuria was observed in one patient. No grade 4 acute urinary toxicity was observed. Acute rectal toxicities consisted mostly of pain and bleeding with defecation. No grade 3 or 4 acute rectal toxicities were observed.

**Table 3:** Acute and late adverse events

	Grade 1	Grade 2	Grade 3	Grade 4
Acute toxicity^a^				
Urinary	107	41	1	0
Rectal	57	14	0	0
Late toxicity^b^				
Urinary	111	52	10	0
Rectal	115	9	6	0

^a^Adverse events occurred within 90 days. Graded based on the National Cancer Institute Common Toxicity Criteria version 2

^b^Adverse events occurred after 91 days. Graded based on the National Cancer Institute Common Toxicity Criteria version 2

**Fig. 3:**
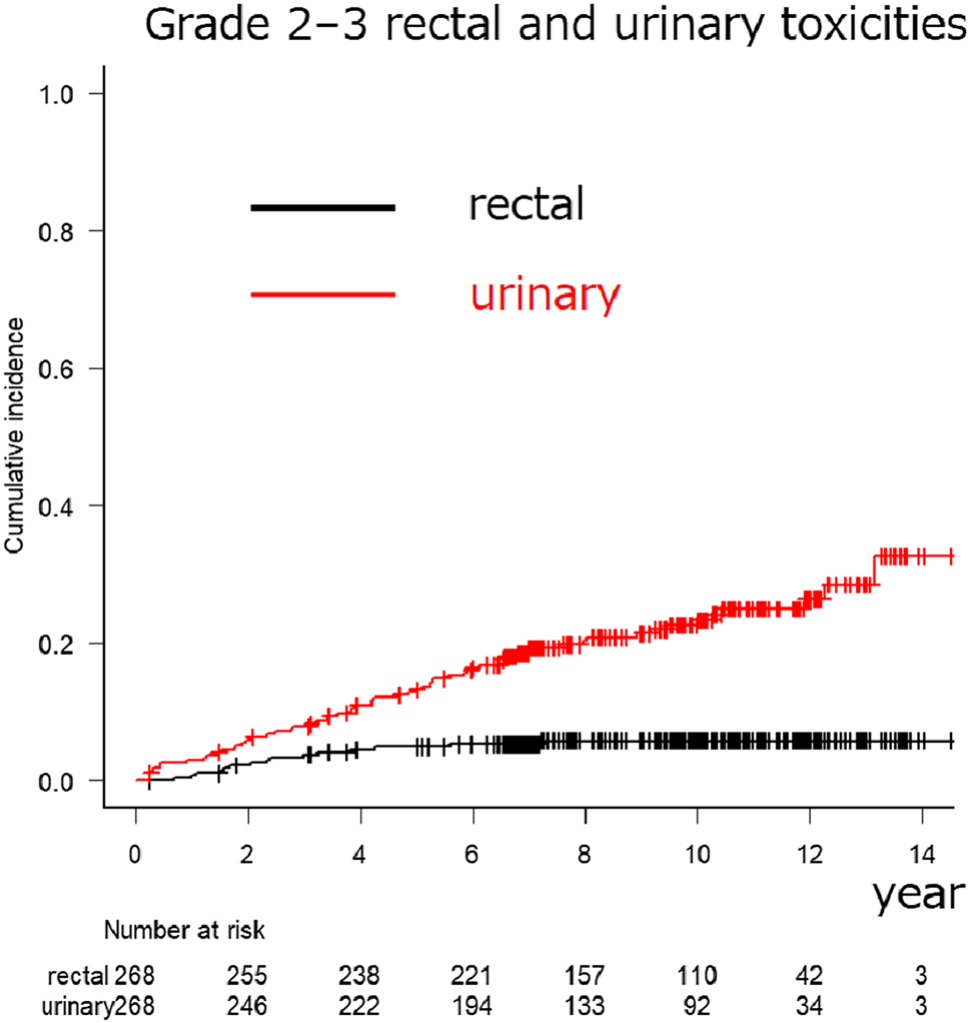
Cumulative incidence rates of grade 2–3 late genitourinary and gastrointestinal toxicities after intensity-modulated radiation therapy

The estimated cumulative incidence rates of grade ≥ 2 late urinary and ≥ 2 late rectal toxicities were 13.3% (95% CI 9.5–17.7) and 4.9% (95% CI 2.7–8.0) at 5 years and 22.6% (95% CI 17.5–28.1) and 5.8% (95% CI 3.4–9.1) at 10 years, respectively (Fig. 3). The majority of late toxicities with urinary or rectal bleeding were transient and improved with time. No grade 4 late toxicity was observed.

### Univariate and multivariable analyses of prognostic factors

According to UVA, iPSA (> 30 vs. ≤ 20 ng/mL; hazard ratio[HR] 2.655; 95% CI 1.786–3.946; *p* < 0.001), T stage (T3b–4 vs. T1–2; HR 2.223; 95% CI 1.465–3.373; *p* < 0.001) and ≥ 5 cores with a GS sum of 8–10 (HR 2.515; 95% CI 1.664–3.8; *p* < 0.001) were significant prognostic factors for BF, and GS sum ( ≥ 8; HR 2.103; 95% CI 1.116–3.964; *p* = 0.022), T stage (T3b–4 vs. T1–2; HR 2.199; 95% CI 1.209–3.999; *p* = 0.0098) and ≥ 5 cores with a GS sum of 8–10 (HR 2.846; 95% CI 1.557–5.204; *p* < 0.001) were significant prognostic factors for CF (Table 4). Among these, according to MVA, iPSA (> 30 vs. ≤ 20 ng/mL; HR 3.172; 95% CI 1.858–5.418; *p* < 0.001, 20–30 vs. ≤ 20 ng/mL; HR 2.165; 95% CI 1.195–3.923; *p* = 0.011) and T stage (T3b–4 vs. T1–2; HR 1.902; 95% CI 1.02–3.548; *p* = 0.043) were significant prognostic factors for BF (Table 4).

**Table 4:** Univariate and multivariable analyses of risk factors for biochemical failure, and clinical failure

Factor	Univariate			Multivariable		
	HR	95% CI	*p*	HR	95% CI	*p*
Biochemical failure						
GS sum; ≥ 8 vs. ≤ 7	1.478	0.9817–2.226	0.061	1.447	0.8568–2.442	0.17
iPSA;						
20–30 vs. ≤ 20 ng/mL	1.141	0.7065–1.842	0.59	2.165	1.195–3.923	0.011
> 30 vs. ≤ 20 ng/mL	2.655	1.786–3.946	< 0.001	3.172	1.858–5.418	< 0.001
T stage;						
T3a vs. T1–2	0.7803	0.524–1.162	0.22	1.017	0.561–1.845	0.95
T3b–4 vs. T1–2	2.223	1.465–3.373	< 0.001	1.902	1.02–3.548	0.043
Cores with GS sum 8–10; ≥ 5 vs ≤ 4	2.515	1.664–3.8	< 0.001	1.262	0.6974–2.283	0.44
Prescription dose; 74 Gy vs 78 Gy	0.7679	0.4577–1.288	0.32	0.975	0.5704–1.666	0.93
Clinical failure						
GS sum; ≥ 8 vs. ≤ 7	2.103	1.116–3.964	0.022	1.769	0.8323–3.761	0.14
iPSA;						
20–30 vs. ≤ 20 ng/mL	0.7025	0.3158–1.563	0.39	0.7985	0.309–2.063	0.64
> 30 vs. ≤ 20 ng/mL	1.606	0.8922–2.89	0.11	1.126	0.5344–2.373	0.75
T stage;						
T3a vs. T1–2	0.7314	0.4052–1.32	0.3	1.029	0.4429–2.39	0.95
T3b–4 vs. T1–2	2.199	1.209–3.999	0.0098	1.908	0.8119–4.484	0.14
Cores with GS sum 8–10; ≥ 5 vs ≤ 4	2.846	1.557–5.204	< 0.001	1.821	0.8307–3.992	0.13
Prescription dose; 74 Gy vs 78 Gy	0.4802	0.1913–1.205	0.12	0.5038	0.1937–1.31	0.16

*HR* hazard ratio, *95% CI* 95% confidence interval, *GS* Gleason score, *iPSA* pretreatment prostate-specific antigen

## Discussion

We retrospectively evaluated the clinical significance of early S-ADT induction based on predetermined timing among patients with unfavorable PCa treated with high-dose IMRT. Our institutional treatment protocol specified high-dose IMRT (78 Gy) combined with short-term NA-ADT alone (CAB of 6 months). No A-ADT was performed because we designed the protocol before the combination of long-term ADT for unfavorable PCa was established as the standard of care. Instead, we initiated S-ADT at an early phase. Despite an absence of long-term A-ADT application, excellent survival outcomes were achieved among the patients with a high risk of PCa-specific mortality (PCSM), as well as favorable long-term disease control. These results support the validity of our treatment strategy for patients with unfavorable PCa.

Based on the results of randomized controlled trials (RCTs) [2-6], addition of long-term ADT to EBRT has been considered a standard treatment approach for unfavorable PCa. However, some issues remain to be resolved. First, these findings were originally based on RCTs that used doses of 70 Gy or lower, which are considered suboptimal in the current IMRT era. Thus, we cannot simply apply these presumptions when combined with high-dose EBRT, because dose escalation significantly improves disease control [11, 12]. Second, the improvement in the PCSM rate achieved with long-term ADT compared with short-term ADT is minimal. In the EORTC 22,961 trial, comparing the benefits of short-term (6 months) and long-term (3 years) ADT combined with 3D-CRT using 70 Gy, the improvement in PCSM was only 1.5% [3]. Similarly, in TROG 03.04 (6 vs. 18 months ± zoledonic acid), long-term ADT failed to improve the OS [4]. When combined with high-dose EBRT, marked survival benefits of long-term ADT were considered less likely because of the expected further improvement in disease control achieved by dose escalation. For these reasons, the optimal duration of ADT in combination with high-dose EBRT remains unclear. In the current study, excellent survival outcomes were achieved with 6-month NA-ADT. In addition, approximately three-fourths of the HR PCa patients and half of the VHR PCa patients in our cohort maintained long-term disease-free status after IMRT and consequently were spared from unnecessary long-term ADT. Therefore, long-term ADT may be excessive for a substantial number of unfavorable PCa patients when combined with high-dose EBRT; thus, it is crucial to identify the groups who will truly benefit from the addition of long-term ADT, which can potentially cause considerable side effects [13].

Our survival outcomes were comparable with those reported previously in studies of unfavorable PCa, although our cohort consisted of a larger number of patients with highly advanced disease compared with those in previous studies [2-6, 14]. We hypothesize that early S-ADT initiation contributed to our favorable survival outcomes. Several secondary analyses of RCTs and retrospective studies have indicated that early initiation of S-ADT results in superior survival outcomes compared with delayed initiation [7, 8]. Mahal et al. reported that among patients who developed BF following definitive EBRT alone or in combination with short-term ADT, the PCSM rate was significantly lower in patients who initiated S-ADT with a PSA level ≤ 12 ng/mL (early S-ADT) than in those with a PSA level > 12 ng/mL (delayed S-ADT) (adjusted HR 8.84; 95% CI 1.99–39.27; *p* = 0.004) [7]. Similarly, Shipley et al. reported a significant improvement in the PCSS rate in patients with a PSA level ≤ 20 ng/mL compared with those with a PSA level > 20 ng/mL at the initiation of S-ADT, among patients who developed BF after EBRT alone or in combination with short-term NA-ADT [8]. However, because the timing of previous S-ADT studies was not predetermined and was based on the physician’s discretion, it is difficult to evaluate the true benefit of S-ADT due to biases, such as increasing PSA velocity or differences in treatment policies among physicians. In the present study, the trigger PSA level for S-ADT initiation was set much lower than the cutoff PSA level of previous studies (trigger level 4.0 ng/mL). Although we delayed S-ADT initiation after BF to eliminate cases with false failures such as PSA bounce, S-ADT was subsequently initiated in most of the patients who developed BF due to continuous PSA elevation (89.7%). This implies that much earlier S-ADT induction, such as at the time of BF, may be more appropriate. Consistent with this, immediate addition of S-ADT after BF outperformed delayed addition in terms of OS in patients with relapsed PSA or noncurable PCa (unadjusted HR 0.55; 95% CI 0.3–1.00; *p* = 0.05) in the TROG 03.06 trial [15]. Logically, if we lowered the trigger PSA levels for initiating S-ADT, it would be almost synonymous with selectively adding long-term A-ADT to patients with a higher risk of PCSM. Therefore, early S-ADT combined with short-term NA-ADT may be a promising alternative to uniform application of long-term A-ADT when combined with high-dose IMRT, although the proper timing of S-ADT initiation should be further investigated, especially in the setting of prospective trials.

Our study has several limitations. This was a retrospective study at single institution, although it reported the outcomes in a large cohort treated with a predetermined protocol and the data collection was based on prospectively maintained databases. Furthermore, because our patient group was Japanese, our findings may not directly apply to other ethnic groups because of the reported differences in hormone sensitivities [16]. Nevertheless, we believe that our study provides baseline data on unfavorable PCa treated with high-dose IMRT combined with short-term NA-ADT, the present study described long-term outcomes utilizing the large cohort of Japanese patients treated under a predetermined uniform treatment policy.

In conclusion, high-dose IMRT combined with short-term NA-ADT resulted in long-term disease-free outcomes, with acceptable morbidities, among approximately three-fourths of the HR PCa patients and nearly half of the VHR PCa patients. Moreover, patients with disease failure were rescued by early initiation of S-ADT, with excellent survival outcomes. This approach may therefore be a promising alternative to the uniform provision of long-term A-ADT, although prospective trials are warranted to confirm these findings.

## References

[1] National Comprehensive Cancer Network (2018) NCCN Guidelines; prostate cancer version 2.2018. In: The category of prostate cancer. https://www.nccn.org/professionals/physician_gls/default.aspx#site. Accessed 8 March 2018

[2] Tosco L, Briganti A, D'Amico AV (2019). Systematic review of systemic therapies and therapeutic combinations with local treatments for high-risk localized prostate cancer. Eur Urol.

[3] Bolla M, de Reijke TM, Van Tienhoven G (2009). Duration of androgen suppression in the treatment of prostate cancer. N Engl J Med.

[4] Denham JW, Joseph D, Lamb DS (2019). Short-term androgen suppression and radiotherapy versus intermediate-term androgen suppression and radiotherapy, with or without zoledronic acid, in men with locally advanced prostate cancer (TROG 03.04 RADAR): 10-year results from a randomised, phase 3, factorial trial. Lancet Oncol.

[5] Horwitz EM, Bae K, Hanks GE (2008). Ten-year follow-up of radiation therapy oncology group protocol 92-02: a phase III trial of the duration of elective androgen deprivation in locally advanced prostate cancer. J Clin Oncol.

[6] Nabid A, Carrier N, Martin AG (2018). Duration of androgen deprivation therapy in high-risk prostate cancer: a randomized phase III trial. Eur Urol.

[7] Mahal BA, Chen MH, Renshaw AA (2018). Early versus delayed initiation of salvage androgen deprivation therapy and risk of prostate cancer-specific mortality. J Natl Compr Cancer Netw.

[8] Shipley WU, Desilvio M, Pilepich MV (2006). Early initiation of salvage hormone therapy influences survival in patients who failed initial radiation for locally advanced prostate cancer: a secondary analysis of RTOG protocol 86-10. Int J Radiat Oncol Biol Phys.

[9] Norihisa Y, Mizowaki T, Takayama K (2012). Detailed dosimetric evaluation of intensity-modulated radiation therapy plans created for stage C prostate cancer based on a planning protocol. Int J Clin Oncol.

[10] Roach M, Hanks G, Thames H (2006). Defining biochemical failure following radiotherapy with or without hormonal therapy in men with clinically localized prostate cancer: recommendations of the RTOG-ASTRO Phoenix Consensus Conference. Int J Radiat Oncol Biol Phys.

[11] Vogelius IR, Bentzen SM (2018). Dose response and fractionation sensitivity of prostate cancer after external beam radiation therapy: a meta-analysis of randomized trials. Int J Radiat Oncol Biol Phys.

[12] Zaorsky NG, Palmer JD, Hurwitz MD (2015). What is the ideal radiotherapy dose to treat prostate cancer? A meta-analysis of biologically equivalent dose escalation. Radiother Oncol.

[13] Haque R, UlcickasYood M, Xu X (2017). Cardiovascular disease risk and androgen deprivation therapy in patients with localised prostate cancer: a prospective cohort study. Br J Cancer.

[14] Zapatero A, Guerrero A, Maldonado X (2015). High-dose radiotherapy with short-term or long-term androgen deprivation in localised prostate cancer (DART01/05 GICOR): a randomised, controlled, phase 3 trial. Lancet Oncol.

[15] Duchesne GM, Woo HH, Bassett JK (2016). Timing of androgen-deprivation therapy in patients with prostate cancer with a rising PSA (TROG 03.06 and VCOG PR 01–03 [TOAD]): a randomised, multicentre, non-blinded, phase 3 trial. Lancet Oncol.

[16] Cooperberg MR, Hinotsu S, Namiki M (2016). Trans-Pacific variation in outcomes for men treated with primary androgen-deprivation therapy (ADT) for prostate cancer. BJU Int.

